# Clinical and laboratory key performance indicators in IVF: A consensus between the Italian Society of Fertility and Sterility and Reproductive Medicine (SIFES-MR) and the Italian Society of Embryology, Reproduction and Research (SIERR)

**DOI:** 10.1007/s10815-023-02792-1

**Published:** 2023-04-24

**Authors:** Alberto Vaiarelli, Carlotta Zacà, Valentina Spadoni, Danilo Cimadomo, Alessandro Conforti, Carlo Alviggi, Roberto Palermo, Carlo Bulletti, Lucia De Santis, Valerio Pisaturo, Vincenzo Vigiliano, Giulia Scaravelli, Filippo Maria Ubaldi, Andrea Borini

**Affiliations:** 1 GeneraLife IVF, Clinica Valle Giulia, Via G. De Notaris, 2B, Rome, Italy; 2 9.Baby, GeneraLife IVF, Bologna, Italy; 3 Department of Neuroscience, Reproductive Science and Odontostomatology, University of Naples Federico II, Naples, Italy; 4 Department of Public Health, Federico II University, Naples, Italy; 5 Unità Di Procreazione Medicalmente Assistita, Centro A.M.B.R.A., Palermo, Italy; 6 Department of Obstetrics, Gynecology and Reproductive Science, Yale University, New Haven, CT USA; 7 Scientific Partner of Incintas Therapeutics, New Haven, CT USA; 8 Obstetrics and Gynaecology Unit, IRCCS San Raffaele Scientific Institute, Vita-Salute San Raffaele University, Milan, Italy; 9 Fondazione IRCCS, Ca’ Granda, Ospedale Maggiore, Policlinico Di Milano, Milan, Italy; 10 ART Italian National Register, National Center for Diseases Prevention and Health Promotion, National Health Institute, Rome, Italy

**Keywords:** Key performance indicators, Consensus meeting, Quality improvement, Assisted reproductive technology, Performance indicators, Recommendations

## Abstract

**Purpose:**

Infertility is increasing worldwide, and many couples seek IVF. Clinical management and laboratory work are fundamental in the IVF journey. Therefore, the definition of reliable key performance indicators (KPIs) based on clinical and laboratory parameters, is essential for internal quality control (IQC). Laboratory performance indicators have been identified and a first attempt to also determine clinical ones has been recently published. However, more detailed indicators are required.

**Methods:**

An Italian group of experts in Reproductive Medicine from both public and private clinics on behalf of SIFES-MR and SIERR was established to define IVF indicators to monitor clinical performance.

**Results:**

The working group built a consensus on a list of KPIs, performance indicators (PIs) and recommendation indicators (RIs). When deemed necessary, the reference population was stratified by woman age, response to ovarian stimulation and adoption of preimplantation genetic testing for aneuploidies (PGT-A). Each indicator was scored with a value from 1 to 5 and a weighted average formula – considering all the suggested parameters—was defined. This formula generates a center performance score, indicating low, average, good, or excellent performance.

**Conclusion:**

This study is intended to provide KPIs, PIs and RIs that encompass several essential aspects of a modern IVF clinic, including quality control and constant monitoring of clinical and embryological features. These indicators could be used to assess the quality of each center with the aim of improving efficacy and efficiency in IVF.

**Supplementary Information:**

The online version contains supplementary material available at 10.1007/s10815-023-02792-1.

## Introduction

Infertility is a widely spread condition increasing over time and the number of infertile couples seeking Assisted Reproductive Technology (ART) increased from 5 to 10% per year [1]. Worldwide, 90 million couples experience infertility [2]. The latest data indicate that in most European countries an average of 2.6% births are conceived through ART per year [3]. Moreover, while in the United States ART accounted for slightly less than 2.1% of births in 2019 [4], in Italy 11,305 children were born, representing 2.7% of the total number of Italian births in 2020 (404,892 live births, https://www.iss.it/rpma-dati-registro).

In Italy, the number of births decrease constantly, with a drop under 400,000 births registered in 2021. Treating infertility with ART is a complex process including clinical work and laboratory procedures [5]. ART involves different steps, such as ovarian stimulation (OS), oocyte pick-up (OPU), oocytes fertilization, embryo culture, and/or cryopreservation in the laboratory, and intrauterine embryo transfer (ET), leading to implantation, pregnancy and possibly the birth of a healthy child [3, 6, 7].

The advances in IVF such as blastocyst culture, aneuploidy testing on trophectoderm biopsies, vitrification, cycle segmentation and the chance to use unconventional stimulation protocols, deeply changed the approach in managing infertile couples [8]. Clinical management of infertile couples and ART laboratory impact the overall treatment success. However, all the progresses to date, from blastocyst culture to vitrification, are closely linked to the efficient management of infertile patients from their first consultation until ET. Careful monitoring of IVF efficiency and fertility care quality throughout the journey is essential. Currently, the main goal of IVF is obtaining a healthy baby in a short timeframe and with the least possible reproductive risks. According to the world health organization (WHO), indeed, high quality fertility care should grant the individuals and the couples with their the right to establish a family (https://www.who.int/news-room/fact-sheets/detail/infertility). Therefore, the identification of key performance indicators score (KPIs) and/or Performance Indicators (PIs) based on clinical and laboratory parameters is important to quantitatively and qualitatively measure each center performance and to guide the internal quality control (IQC) in ART [9]. PIs are elements used to quantify specific achievements, monitor, and constantly improve the results, as required by the QMS (quality management system). Both KPIs and PIs must be measurable, reproducible, consistent, and appropriate to define the effectiveness and safety of care. They must be also agreed upon by a consensus of experts [10]. Each IVF center, both private and public ones, should constantly monitor their performance indexes to assess the quality of the procedures. Nonetheless, the relevant indexes are highly complex and tend to be difficult to monitor. Moreover, the lack of standardization of the parameters used limits both outcomes’ monitoring and overall performance. The standardization of the parameters would substantially improve IVF procedures and enable the comparison of the results across centers.

Laboratory indicators have been already identified during two international consensuses regarding: (i) a minimum list of indicators, (ii) their definitions (including inclusion/exclusion criteria and calculation formulae), and (iii) values for each KPI (minimum ‘competence’ limit and ‘aspirational goal’ benchmark) [9, 11]. The first attempt to determine clinical indicators in IVF, instead, was recently published in the Maribor Consensus [5]. However, many aspects remain unclear and demand further in-depth analysis. Specifically, the Maribor consensus considered only women < 40 years as reference population, without a further stratification indicative of different patients’ prognosis. This is crucial, though, considering that different classifications exist to better stratify poor prognosis patients, especially in case of women > 35 years, when a dramatical decline of prognosis is observed on a yearly basis [12–14]. Consequently, specific indicators should be developed based on a more detailed age stratification. Other unsolved issues are related with the lack of specific indicators of ovarian response and concerning same couple or third-party reproduction. Finally, no indicators were proposed to assess the first steps of fertility care (infertility work-up or time between the first consultation and treatment decision making). Considering all these issues, the aim of this Consensus was to overcome them by developing more detailed IVF indicators in a collaboration between clinicians and embryologists on behalf of the Italian Society of Fertility, Sterility and Reproductive Medicine (SIFES-MR) and the Italian Society of Embryology, Reproduction and Research (SIERR). At last, we defined a methodology to outline a measurable center performance score (CPS) at each IVF clinic, which might be useful as a self-assessment IQC tool.

## Methods

**Fig. 1:**
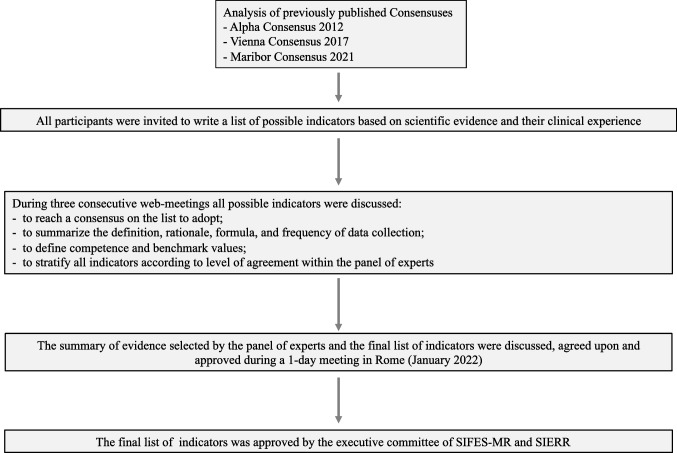
Overview of the workflow adopted to build the SIFES-MR and SIERR Consensus on the performance indicators for IVF clinical practice

The scientific board was composed of experts in Reproductive Medicine working at Italian IVF centers. They were representative of three Italian geographical areas (i.e., northern, central, and southern) and active in either public or private clinics. Both clinicians and embryologists were involved. Also, two members of the ART Italian National Register were involved. Figure 1 summarized the workflow of this Consensus.

AV, CZ, and VS analyzed the three published international consensuses to summarize and identify their inherent limitations. All experts were then invited to list the indicators adopted as part of their clinical practice to assess and improve the quality of their IVF setting, based on scientific evidence and clinical experience. Three consecutive web meetings were organized to discuss the KPIs aiming at (i) reaching a consensus on the list of suggested indicators; (ii) summarizing definition, rationale, formula, and frequency of data collection; and (iii) define competence and benchmark values. The summary of evidence selected by the expert panel and the final list of KPIs/PIs/RIs were discussed and agreed upon during a one-day meeting held in Rome in January 2022. During the consensus meeting, the results of internal surveys, scientific evidence and personal clinical experience were integrated into this document by the experts to finally find a Consensus on the recommended list of competence and benchmark values. The list of indicators was finally shared with all members of SIFES-MR and SIERR executive committees for their comments. Three different levels of agreement were outlined within the panel of experts:

1. KPIs with high agreement (> 80%).
2. PIs with medium agreement (40–80%).
3. RIs with poor agreement (< 40%).

The panel ranked these indicators based on the latest published level of evidence. For each indicator proposed and shared among the panel, the following information were considered: definition, rationale, formula, data sources, strengths and weaknesses, frequency of data collection, and limitations. Moreover, minimum expected or competence values (i.e., values that any laboratory should be able to achieve) and benchmark values (i.e., values that shall represent the best practice goal) were included for each indicator based on both the current literature and personal experience.

To identify more applicable and realistic indicators, the reference population was further stratified based on woman age (≤ 34 years, 35–39 years, ≥ 40 years) and ovarian response (expected poor, normal, and high responders). The definition of poor, normal and high responders is based on the number of oocytes retrieved. While poor responders are women collecting less than 4 oocytes [15], high responders collect more than 15 oocytes [16]. Normal responders, instead, collect 10–15 oocytes. The stratification was carried out only when deemed necessary for certain KPIs. When the indicators are not stratified in terms of age or ovarian response, we simply referred to “all patients” or “reference population”.

## Results

*KPIs: Statements with high agreement (*> *80%) (*Table 1*)*

- *1. Cycle cancellation rate (before OPU) (%CCR)*
  - **Definition:** Cycle cancellation rate was defined as treatment discontinuation before OPU.
  - **Formula:** Number of cycles cancelled before OPU / Number of started cycles (i.e., ovarian stimulation initiated)
  - **Competence and benchmark values:**
    - Minimum Expected: Poor responders ≤ 30%; Normal and Hyper responders ≤ 3%
    - Best Practice Goal: Poor responders ≤ 10%; Normal and Hyper Responders ≤ 0.5%
  - **Frequency of analysis:** 3 months or 100 cycles, whichever comes first.
  - **Population:** Poor, normal and hyper responders
  - **Rationale:** Cancellation of an IVF cycle is an unexpected outcome that can occur prior to or after OPU. The cycle cancellation before OPU was agreed upon as a relevant parameter to assess ovarian stimulation performance. Overall cycle cancellation before OPU is estimated as 7.9% [17, 18] due to poor or excessive response to ovarian stimulation, premature ovulation, or errors in assuming the medications. This parameter is more accurate than cycle cancellation before embryo transfer (ET) as the latter can be influenced by many factors such as local reimbursement policies, patient preferences, or IVF strategies (freeze all, extended embryo culture, and PGT-A) [17]. Indeed, the reported cancellation rates are likely to underestimate the true ones [19].
- *2. Late Follicle-to-Oocytes index (FOI):*
  - **Definition:** The FOI assesses the consistency between the pool of antral follicles at the beginning of ovarian stimulation (up to the fifth day of stimulation) and the number of oocytes retrieved at OPU [20]. Late FOI was proposed by the panel to reduce the expected inter-cycle and inter-observer antral follicle count variability in routine clinical practice.
  - **Formula:** Number of oocytes retrieved at OPU / number of antral follicles at the beginning of ovarian stimulation [21].
  - **Competence and benchmark values:**
    - Minimum Expected: ≥ 50%
    - Best Practice Goal: ≥ 80%
  - **Frequency of analysis:** Monthly or every 100 cycles, whichever comes first.
  - **Population:** Reference population
  - **Rationale:** Ovarian stimulation is essential in ART. The prediction of ovarian response is crucial for an optimal and individualized management [20]. FOI was described elsewhere and used to measure the ovarian sensitivity to exogenous gonadotropins [15, 20]. The aim was to verify whether clinicians can adequately conduct ovarian stimulation. In fact, comparing the number of oocytes retrieved to the cohort of follicles matured during ovarian stimulation is valuable to understand if starting dose, triggering and oocyte retrieval were properly defined and conducted. The pathogenesis of hypo-response to gonadotropin stimulation (ovarian resistance) seems associated with genetic or environmental factors, asynchronous follicular development or technical issues involving triggering for final oocytes maturation or OPU [20, 22, 23]. FOI should be used to identify the subset of hypo-responders and determine if the ovarian reserve was adequately exploited [22].
- *3. Proportion of MII oocytes at ICSI (% MII)*
  - **Definition:** Proportion of mature oocytes available for ICSI
  - **Formula:** Number of metaphase-II (MII) oocytes at ICSI / number of cumulus oocyte complexes (COCs) retrieved
  - **Competence and benchmark values:**
    - Minimum Expected: ≥ 75%
    - Best Practice Goal: ≥ 90%
  - **Frequency of analysis:** Monthly or every 100 cycles, whichever comes first.
  - **Population:** Reference population
  - **Rationale:** All steps are important in IVF treatment to maximize the chance of success. Induction of final oocyte maturation is one of the most crucial steps. Indeed, choosing the right trigger is pivotal [24]. Suboptimal oocytes yield/high immaturity rate can be due to (i) low hCG intra-follicular levels (high BMI; injection errors); (ii) < 35 h between injection and OPU; (iii) aspirated follicles < 14 mm; (iv) LH receptor deficiency. Of note, BMI is inversely related to intra-follicular hCG concentration. For all these reasons, the proportion of MII oocytes at ICSI was identified as a RI in both the Vienna and Maribor consensuses. This is a proxy indication of the effectiveness of ovarian stimulation [5], mirroring factors that influence the number of oocytes available for fertilization. Values outside the normal range must prompt a review of any changes in ovarian stimulation, triggering, or follicle aspiration practices, as the proportion of MII oocytes could affect cumulative reproductive outcomes by affecting developmental competence, clinical pregnancy, and live birth rates [25, 26].
- *4. Complication rate after OPU (% Co-OPU)*
  - **Definition:** Complications of OPU include bleeding (severe vaginal, intra-abdominal, or intra-peritoneal bleeding), infection (pelvic or ovarian abscess, pelvic infections), severe pain, or injury of pelvic structures.
  - *Formula:* Number of complications (any) that require an (additional) medical intervention or hospital admission (OHSS excluded) / Number of OPUs
  - *Competence and benchmark values:*
    - Minimum Expected: ≤ 0.5%
    - Best Practice Goal: ≤ 0.1%
  - *Frequency of analysis:* 3 months or every 50 cycles, whichever comes first.
  - *Population:* Reference population
  - *Rationale:* Ultrasound-guided transvaginal route (US-TV) is the most common approach used to collect oocytes during IVF [27]. The oocyte retrieval procedure can be considered safe, although patients and physicians should recognize it is not without risks. The complication rate per OPU has been calculated to around 0.4% overall. A surgical procedure is needed in few cases (0.1% per retrieval). Vaginal bleeding is the most common OPU complication, with a reported prevalence ranging 0.01% to 18.8%. This high difference is imputable to an inconsistent definition of vaginal bleeding [28–30]. OPU complications were consistently fewer when the operators had performed ≥ 250 procedures. Risk factors for these complications are a high number of oocytes retrieved, a long duration of the procedure, surgeon inexperience, younger patients with low BMI, history of abdominal or pelvic surgery, and previous pelvic inflammatory diseases [28]. Rarer complications, described as case reports, are ureterovaginal fistulas, pseudo-aneurysm of the iliac artery, ureteral injury, bladder injury with hematuria, ovarian torsion, and ovarian abscess [29, 30]. Complications related to sedation or anesthesia have also been reported but are not considered a relevant PI for clinical practice in ART.
- *5. ICSI Fertilization rate*
  - **Definition:** The proportion of injected oocytes with 2 pronuclei (PN) and 2 polar bodies (PB) the day after injection.
  - **Formula:** Number of oocytes with 2PN and 2 PB / number of MII oocytes injected
  - **Competence and benchmark values:**
    - Minimum Expected: ≥ 65%
    - Best Practice Goal: ≥ 80%
  - **Frequency of analysis:** Monthly or every 100 cycles, whichever comes first.
  - **Population:** Reference population excluding cases where reduced fertilization rates are anticipated, including in-vitro matured or artificially-activated oocytes, and cases of severe male factor [31]. Severe male factor infertility encompasses severe oligozoospermia (< 5 × 10^6^ sperms per ml of ejaculate), cryptozoospermia, and absence of spermatozoa in the ejaculate that requires surgical retrieval [32]. Thawed/warmed oocytes cycles are also excluded [33].
  - **Rationale:** Normal fertilization rate is considered a relevant parameter to assess ovarian stimulation performance [34]. This is an essential KPI to evaluate the introduction of a technique or process, establishing minimum standards for proficiency, monitoring ongoing performance within a quality management system (QMS; for IQC or external quality assurance [EQA]), benchmarking and quality improvement. It has been adopted as a KPI of the IVF laboratory to assess both operator and gamete competence [11]. It is a commonly reported and effective indicator, informative of gamete quality and/or operator skills. Of note, ICSI 2PN rate does depend on the various criteria adopted to opt for ICSI, which can represent a weakness. Nevertheless, several studies showed the importance of ICSI fertilization as a KPI, which can impact on clinical outcomes. A recent retrospective study [35], indeed, showed fertilization rates significantly associated with the clinical outcome. Moreover, Rosen et al. [36] conducted a study involving 603 couples undergoing IVF and demonstrated that fertilization rate is a strong predictor of implantation [36]. Recently, Scaravelli et al. [37] as well, demonstrated a positive association between fertilization rate and cumulative live birth rate (CLBR) across more than 9,000 cycles in the Italian ART register, thereby further supporting the predictive power of this parameter [37]. This association stood also after correction in a multivariate logistic regression.
- *6. Proportion of embryos with ≥ 8 cells on day 3*
  - **Definition:** The proportion of embryos with at least 8 cells on day 3 (measured at 68 ± 1 h post insemination).
  - **Formula:** Number of embryos on day 3 with at least 8 cells / Number of normally fertilized oocytes (i.e., oocytes with 2PN and 2 PB on day 1)
  - **Competence and benchmark values:**
    - Minimum Expected: ≥ 45%
    - Best Practice Goal: ≥ 70%
  - **Frequency of analysis:** Monthly or every 100 cycles, whichever comes first.
  - **Population:** Reference population.
  - **Rationale:** This KPI reflects the ability of the culture system to support cleavage stage development according to the expected developmental rate and the quality and viability of embryos, especially for day 2 or day 3 transfers [11]. In evaluating this indicator, possible confounders are the timing of laboratory observations and the type of culture media used. Although culture conditions could influence embryo development, day 3 embryo development rate is an important indicator because it reflects the overall laboratory performance. Of note, a recent study showed that the blastocyst formation rate is associated with the number of cells in day 3 and in particular with a higher proportion of good-quality blastocysts in the > 8 cell group [38].
- *7. Total blastocyst development rate*
  - **Definition:** The total blastocyst development rate is defined as the proportion of 2PN zygotes that develop to the blastocyst stage up to 168 h post insemination [9].
  - **Formula:** Number of blastocysts obtained / Number of normally fertilized oocytes (i.e., oocytes with 2PN and 2 PB on day 1)
  - **Competence and benchmark values:**
    - Minimum Expected: ≥ 45% (≤ 34–39 yr); ≥ 35% (≥ 40 years)
    - Best Practice Goal: ≥ 65% (≤ 34–39 yr); ≥ 55% (≥ 40 years)
  - **Frequency of analysis:** Monthly or every 100 cycles, whichever comes first.
  - **Population:** Reference population stratified according to the following age ranges (i) ≤ 34–39 yr; (ii) ≥ 40 years.
  - **Rationale:** Total blastocyst development rate is considered important because it reflects the efficiency of the whole culture system [11]. In particular, it estimates its ability to support blastocyst formation from fertilized oocytes until formation of blastocoele cavity, inner cell mass, and trophectoderm, and indicates embryo viability. It should be noted that this definition only considers blastocyst formation, but not its stage (day 5–7) or quality. Moreover, confounders exist such as timing of observation, culture medium, and culture conditions. This parameter was chosen because several studies emphasized the importance of these laboratory data in influencing clinical results [34, 39, 40]. We also established an assessment of the data by stratifying for maternal age as some studies reported that quantity and quality of blastocysts formed are independent from the number of collected oocytes, but negatively associated with female age [39]. In addition, several authors showed a maternal age-dependent effect on embryo development emerging only at the blastocyst stage [34, 41, 42]. This may derive from several molecular, biochemical, and cellular oocyte dysfunctions imputable to aging [43]. Vassena et al. suggested that the differences observed at the blastocyst stage may result from alterations in the embryonic genome activation processes [44]. Previous Consensuses suggested the “useable” blastocyst rate as a KPI. However, SIFES-MR and SIERR panel of experts reckons that the term “useable” relies on subjective and inconsistent evaluations [45] and heterogenous clinical policies (e.g., day 7 culture being conducted or not [46]), thereby limiting the reproducibility of “useable blastocyst rate” as KPI. The “total blastocyst development rate” is instead less dependent on center-specific practice and expertise. A “blastocyst” is defined as any embryo that has completed blastocoel formation and whose inner cell mass is clearly visible (independently from its morphological quality). This would correspond to an embryo at the tB defined according to the ESHRE time lapse technology working group as the “last frame before zona [pellucida] starts to thin” [47].
- *8. Clinical pregnancy rate (% CPR)*
  - **Definition:** Clinical pregnancy is defined as a pregnancy confirmed on ultrasonographic visualization of one or more gestational sacs with fetal heartbeat or definitive clinical signs of pregnancy [48].
  - **Formula:** Number of pregnancies (diagnosed by ultrasonographic visualization of one or more gestational sacs with fetal heartbeat or definitive clinical signs of pregnancy) / number of first embryo transfers (either fresh or frozen/vitrified).
  - **Competence and benchmark values:**
    - Minimum Expected: ≥ 30% (≤ 34 years); ≥ 20% (35–39 years); ≥ 10% (≥ 40 years) [14].
    - Best Practice Goal: ≥ 40% (≤ 34 years); ≥ 30% (35–39 years); ≥ 20% (≥ 40 years) [14]
    - PGT-A Cycle:
      - Minimum Expected: ≥ 45%
      - Best Practice Goal: ≥ 60%
  - **Frequency of analysis:** 3 months or every 100 cycles, whichever comes first.
  - **Population:** Reference population stratified according to the following age range for untested embryo transfers: (i) ≤ 34 years; (ii) 35–39 years; (iii) ≥ 40 years. In PGT-A cycles, no age stratification is entailed for euploid embryo transfers.
  - **Rationale:** Only first embryo transfers should be considered because (i) they mostly entail better quality and faster developing embryos, and (ii) to prevent the influence of poor prognosis patients facing multiple failures and therefore undergoing multiple transfers of possibly progressively poorer quality and slower growing embryos [49–51]. The estimates provided here represent an overall expected outcome entailing either untested cleavage stage or blastocyst transfers. Nevertheless, this panel of experts agreed that the latter might involve better results per transfer [52]. Euploid blastocysts transferred in the context of PGT-A cycles should result in CPR per transfer higher than 50% almost independently from maternal age [53]. A single embryo transfer approach is strongly recommended to minimize the establishment of multiple pregnancies subject to significantly increased complications (see next KPI). The panel of experts acknowledges the live birth rate (LBR) as more accurate than the CPR to assess the efficiency per transfer of an IVF center, but also that not all clinics follow-up pregnancies up to live birth. Both CPR and LBR are calculated per first transfer though, therefore overlooking all cycles not reaching this treatment stage. This makes these measures well representative of the efficiency of embryo selection and embryo transfer procedures, but poorly representative of the overall performance of an IVF center (i.e., efficacy). Cumulative live birth rate (CLBR), instead, is the main clinical measure of success in IVF, comprehensively summarizing the efficacy of each started cycle (i.e., ovarian stimulation initiated) [54]. Nevertheless, CLBR requires one year or more to be calculated and it cannot be used to assess the performance of an IVF center in the short- or medium-term, therefore also being poorly effective for IQC purposes. It was therefore considered by this Consensus a PI and not a KPI (commented later in the manuscript). CPR and total blastocyst development rate as KPIs may partially compensate for CLBR; however, this indicator should still be calculated every year as a PI.
- *9. Multiple pregnancy rate (% MPR)*
  - **Definition:** A pregnancy with more than one fetus is defined a multiple pregnancy.
  - **Formula:** Number of pregnancies with more than one fetus / number of pregnancies
  - **Competence and benchmark values:**
    - Minimum Expected: ≤ 10%
    - Best Practice Goal: ≤ 5%
  - **Frequency of analysis:** 3 months or every 50 cycles, whichever comes first.
  - **Population:** Reference population (including egg donation cycles).
  - **Rationale:** The prevalence of multiple pregnancy in natural conceptions is ≈1%. Women with a twin pregnancy are 6 times more likely to be hospitalized with complications, especially when of advanced maternal age. Multiple pregnancy is associated with high gestational risks (2–threefold increase versus singletons) including prematurity (17% of all preterm birth; sixfold increase), low birthweight (24% of low birth-weight infants < 2,500 g and 26% of very-low-birth-weight < 1,500 g), hypertensive pregnancy disorders (3–fourfold increase), gestational diabetes, postpartum hemorrhage, premature rupture of the membranes, hyperemesis, severe anemia, operative delivery, neonatal morbidity and high neonatal and infant mortality [55, 56]. Therefore, a single blastocyst transfer is strongly recommended to ensure safety to all infertile couples, and especially in advanced maternal age women undergoing PGT-A or egg donation cycles, to reduce the prevalence of multiple pregnancies [57]. Of note, the risk of multiple pregnancies after single embryo transfer is around 1–2% and no further action can be undertaken to reduce this value at present [58].
- *10. Miscarriage Rate*
  - **Definition:** The number of spontaneous losses of a clinical intrauterine IVF-derived pregnancy.
  - **Formula:** Number of miscarriages / Number of clinical pregnancies
  - **Competence and benchmark values:**
    - Minimum Expected: ≤ 20% (≤ 34 years); ≤ 35% (35–39 years); ≤ 50% (≥ 40 years)
    - Best Practice Goal: ≤ 15% (≤ 34 years); ≤ 25% (35–39 years); ≤ 40% (≥ 40 years)
    - PGT-A cycle:
      - Minimum Expected: ≤ 15%
      - Best Practice Goal: ≤ 10%
  - **Frequency of analysis:** 6 months or every 100 cycles, whichever comes first.
  - **Reference population:** Reference population stratified according to the following age range for untested ETs: (i) ≤ 34 years; (ii) 35–39 years; (iii) ≥ 40 years. In PGT-A cycles, no age stratification is entailed for euploid ETs.
  - **Rationale:** Pregnancy loss after IVF ranges 13–32% [59]. Higher prevalence of pregnancy loss is reported among advanced maternal age women with an average of 35–40% in women older than 42 years [60, 61]. Pregnancy loss also reflects the efficacy of luteal phase support. In addition, considering that 8–10% of miscarriage could be linked to endocrine or metabolic disorders [62], the assessment of these factors could help reducing the risk for this adverse outcome. Pregnancy loss per clinical pregnancy is independent from the embryonic stage in the context of untested embryo transfers [52]. Conversely, euploid blastocyst transfer reduces the risk of miscarriage to 15% or less, independently from maternal age, therefore specific competence and benchmark values apply to PGT-A cycles.
- *11. Rate of cycles with moderate/severe OHSS*
  - **Definition:** OHSS, a complication of fertility treatment, is characterized by vomiting, abdominal pain, clinical ascites, oliguria/anuria, hematocrit > 0.45, hyponatremia (sodium < 135 mmol), hypo-osmolality, hypoproteinemia (serum albumin < 35 g/l; ovarian sizes usually 8–12 cm), thromboembolism, and acute respiratory distress syndrome [63].
  - **Formula:** Number of cycles with moderate or severe OHSS / number of started cycles (i.e., ovarian stimulation initiated)
  - **Competence and benchmark values:**
    - Minimum Expected: ≤ 3%
    - Best Practice Goal: ≤ 0.5%
  - **Limitations:** The definition of OHSS varies across all studies included in a Cochrane meta-analysis [64].
  - **Frequency of analysis:** 6 months or every 100 cycles, whichever comes first.
  - **Population:** Expected normal and hyper responders
  - **Rationale:** High daily dose during ovarian stimulation may increase OHSS prevalence in patients with high ovarian reserve markers, and in modern IVF it is mandatory minimizing its risk. Nonetheless, optimizing ovarian response to stimulation is crucial as well to increase the CLBR per started cycle. Several follicle thresholds have been proposed as critical to predict the occurrence of OHSS, namely 14 follicles > 11 mm for the general population [65] or > 20 follicles > 11 mm for patients without polycystic ovary syndrome (PCOS) or non-poor responder patients [66]. The incidence of severe OHSS reported in clinical studies varies from 2% [65] to almost 9% [67]. In this regard, identification of hyper-responders is very important to reduce OHSS risk. Lately, OHSS prevalence has been significantly reduced via GnRH antagonist protocols. Indeed, GnRH agonist triggering, instead of hCG, and freeze-all (i.e., cycle segmentation policy) represents the most effective workflow to almost eradicate this complication [68]. A correct assessment of the ovarian reserve, along with couple’s clinical history (i.e., previous experience of OHSS), and individualization of medications’ starting dose, are all crucial before starting ovarian stimulation. In our view, OHSS prevalence is critical to assess clinicians’ performance, especially because ART registries to date do not inspect this value nor report its prevalence.

**Table 1:** List of KPIs identified by SIFES-MR and SIERR panel of experts with high agreement (> 80%). To outline more applicable and realistic indicators for some of them, the reference population has been stratified based on maternal age and ovarian response to the stimulation. The table represents an overview of competence and benchmark values for the each KPI along with the suggested frequency of their analysis and a suggested value for the calculation of the center performance score (CPS)

KEY PERFORMANCE INDICATORS	Reference population	Competence Value	Benchmark Value	Suggested frequency of analysis	Suggested Value of this KPI (1 = low importance to 5 = high importance)
1. Cycle Cancellation Rate (before OPU) (% CCR)	a) Poor responders	a) ≤ 30%	a) ≤ 10%	3 months or 100 cycles	3
	b) Normal and Hyper responders	b) ≤ 3%	b) ≤ 0.5%		
2. Late Follicle-to-Oocytes index (FOI)	All patients	≥ 50%	≥ 80%	Monthly or 100 cycles	4
3. Proportion of MII oocytes at ICSI (% MII)	All patients	≥ 75%	≥ 90%	Monthly or 100 cycles	3
4. Complication rate after OPU (% Co-OPU)	All patients	≤ 0.5%	≤ 0.1%	3 months or 50 cycles	2
5. ICSI fertilization rate	All patients *(Except for IVM, SMF, AOA, and vitrified-warmed oocytes)*	≥ 65%	≥ 80%	Monthly or 100 cycles	4
6. Proportion of embryos with ≥ 8 cells on day 3	All patients	≥ 45%	≥ 70%	Monthly or 100 cycles	3
7. Total blastocyst development rate	a) ≤ 34–39 years	a) ≥ 45%	a) ≥ 65%	Monthly or 100 cycles	5
	b) ≥ 40 years	b) ≥ 35%	b) ≥ 55%		
8. Clinical Pregnancy Rate (% CPR)	a) ≤ 34 years	a) ≥ 30%	a) ≥ 40%	3 months or 100 cycles	5
	b) 35–39 years	b) ≥ 20%	b) ≥ 30%		
	c) ≥ 40 years	c) ≥ 10%	c) ≥ 20%		
	d) PGT-A (all patients)	d) ≥ 45%	d) ≥ 60%		
9. Multiple Pregnancy Rate (% MPR)	All patients *(Including egg donation)*	≤ 10%	≤ 5%	3 months or 50 cycles	5
10. Miscarriage rate	a) ≤ 34 years	a) ≤ 20%	a) ≤ 15%	6 months or 100 cycles	3
	b) 35–39 years	b) ≤ 35%	b) ≤ 25%		
	c) ≥ 40 years	c) ≤ 50%	c) ≤ 40%		
	d) PGT-A (all patients)	d) ≤ 15%	d) ≤ 10%		
11. Rate of cycles with moderate-severe OHSS (% OHSS)	All patients	≤ 3%	≤ 0.5%	6 months or 100 cycles	5

*OPU* oocyte pick-up, *OHSS* ovarian hyperstimulation syndrome, *IVM* in vitro maturation, *SMF* severe male factor, *AOA* artificial oocyte activation

*PIs: Statements with medium agreement (40–80%) (*Table 2*)*

**Table 2:** List of PIs identified by SIFES-MR and SIERR panel of experts with medium agreement (40–80%). Competence and benchmark values were proposed for each KPI, along with a suggested frequency for their analysis

PERFORMANCE INDICATORS	*Reference population*	*Competence value*	*Benchmark value*	*Suggested frequency of analysis*
1. IVF fertilization rate	All patients (*except for SMF)*	≥ 60%	≥ 75%	Monthly or 100 cycles
2. Oocytes cryo-survival rate	All patients	≥ 70%	≥ 85%	Monthly or 100 cycles
3. Embryo cryo-survival rate: > 50% blastomeres survived AND 3. Embryo cryo-survival rate: all blastomeres survived	All patients	≥ 80%	≥ 95%	Monthly or 100 cycles
		≥ 70%	≥ 85%	
4. Blastocyst cryo-survival rate	All patients	≥ 90%	≥ 99%	Monthly or 100 cycles
5. Successful biopsy rate	All patients	≥ 95%	≥ 97%	Monthly or 100 cycles
6. Follicular output rate (FORT)	All patients	≥ 40%	≥ 80%	Monthly or 100 cycles
7. Cumulative live birth rate (CLBR)	a) ≤ 34 years	a) ≥ 30%	a) ≥ 40%	Yearly
	b) 35–39 years	b) ≥ 20%	b) ≥ 30%	
	c) ≥ 40 years	c) ≥ 5%	c) ≥ 10%

- *1. IVF fertilization rate*
  - **Definition:** The proportion of oocytes with 2 pronuclei (PN) and 2 polar bodies (PB) the day after conventional IVF.
  - **Formula:** Number of oocytes with 2PN and 2 PB / number of cumulus oocyte complexes
  - **Competence and benchmark values:**
    - Minimum Expected: ≥ 60%
    - Best Practice Goal: ≥ 75%
  - **Frequency of analysis:** Monthly or every 100 cycles, whichever comes first.
  - **Population:** Refence population, excluding severe male factors.

- *2. Oocyte cryo-survival rate*
  - **Definition:** Proportion of morphologically intact oocytes at the time of ICSI after thawing-warming.
  - **Formula:** Number of survived oocytes / number of thawed-warmed oocytes.
  - **Competence and benchmark values:**
    - Minimum Expected: ≥ 70%
    - Best Practice Goal: ≥ 85%
  - **Frequency of analysis:** Monthly or every 100 cycles, whichever comes first.
  - **Population:** Refence population.
- *3. Embryo cryo-survival*
  - **Definition:** Survival was defined as the proportion of thawed-warmed viable embryos with at least 50% blastomeres intact and with all blastomeres intact.
  - **Formula:** Number of thawed-warmed embryos with at least 50% of blastomeres intact / number of thawed-warmed embryos AND Number of thawed-warmed embryos with all blastomeres intact / number of thawed-warmed embryos
  - **Competence and benchmark values:**
    - Minimum Expected: ≥ 80% and ≥ 70%, respectively
    - Best Practice Goal: ≥ 95% and ≥ 85%, respectively
  - **Frequency of analysis:** Monthly or every 100 cycles, whichever comes first.
  - **Population:** Refence population.
- *4. Blastocyst cryo-survival*
  - **Definition:** Blastocyst cryo-survival was defined as at least 75% of cells intact after thawing-warming
  - **Formula:** Number of survived blastocysts / number of thawed-warmed blastocysts.
  - **Competence and benchmark values:**
    - Minimum Expected: ≥ 90%.
    - Best Practice Goal: ≥ 99%.
  - **Frequency of analysis:** Monthly or every 100 cycles, whichever comes first.
  - **Population:** Refence population.
- *5. Successful biopsy rate*
  - **Definition:** Proportion of biopsied samples where DNA is successfully detected.
  - **Formula:** Number biopsies with DNA detected / number of biopsies performed.
  - **Competence and benchmark values:**
    - Minimum Expected: ≥ 95% [69]
    - Best Practice Goal: ≥ 97% [70, 71]
  - **Frequency of analysis:** Monthly or every 100 cycles, whichever comes first.
  - **Population:** Refence population.
- *6. Follicular output rate (FORT)*
  - **Definition:** A measure of the pool of antral follicles at the beginning of ovarian stimulation that become pre-ovulatory follicles at the end [72, 73].
  - **Formula:** Number of pre-ovulatory follicles / late antral follicle count.
  - **Competence and benchmark values:**
    - Minimum Expected: ≥ 40%
    - Best Practice Goal: ≥ 80%
  - **Frequency of analysis:** Monthly or every 100 cycles, whichever comes first [20].
  - **Population:** Refence population.
- *7. Cumulative live birth rate (CLBR)*
  - **Definition:** Started cycles (i.e., ovarian stimulation initiated) that result in the live birth of at least one baby.
  - **Formula:** The number of deliveries with at least one live birth resulting from one started cycle / all cycles in which all embryos are transferred until a delivery occurs or until all embryos are used (including all cycles without transferable embryos obtained as well), whichever occurs first.
  - **Competence and benchmark values:**
    - Minimum Expected: ≥ 30% (≤ 34 years); ≥ 20% (35–39 years); ≥ 5% (≥ 40 years) [74, 75]
    - Best Practice Goal: ≥ 40% (≤ 34 years); ≥ 30% (35–39 years); ≥ 10% (≥ 40 years) [74, 75]
  - **Frequency of analysis:** Yearly.
  - **Population:** Refence population.

### PIs rationale

IVF fertilization is less affected by operators’ performance and clinical policies. Therefore, this indicator was not considered a KPI, like ICSI fertilization rate. Nonetheless, we added it to our list of PIs. When it comes to oocyte cryopreservation procedure, instead, embryologists’ performance is critical. This stage of development indeed is the most delicate and perhaps least tolerant to cryopreservation [8], therefore being exposed to large inter-center variability. Nonetheless, oocyte cryo-survival is adopted for fertility preservation, accumulation strategies, surplus oocytes after OPU, but all these practices are not the routine of an IVF center. On the contrary, embryo cryopreservation is part of the daily IVF practice and should be optimized in every clinic, as it allows for increased CLBR and offers the possibility to reduce multiple pregnancies and OHSS risk [8]. In Europe, the proportion of cryopreserved ETs is growing compared to fresh ones. Overall, it has been estimated that cryopreserved cycles contributed to 32% of the transfers conducted in 2011 [8]. From a technical perspective, vitrification is the most efficient cryopreservation strategy, as this technique significantly increases oocyte and embryo cryo-survival rates compared to slow freezing. Vitrification led to improved clinical outcomes and made both fertility preservation and donor oocyte banks solid options for patients. Furthermore, vitrification allowed for cycle segmentation in IVF to temporally disconnecting the stimulation process from ET, thereby also providing additional time to implement (non-)invasive embryo selection strategies, such as PGT-A, with the aim of identifying euploid embryos with greater chance of implantation per ET. In this regard, successful biopsy rate was included here as an indicator of embryologists’ performance with the biopsy and tubing procedures [11]. In the latest ESHRE PGT Consortium data report at the ESHRE annual meeting held in Milan in 2022 (Monday, July 4th, 11:45—12:45, Silver Room, Session code:1100, Session title: Session 10—Data reporting session, Title: O-041: Data from the ESHRE PGT consortium – year 2020) it was stated that the prevalence of blastocyst biopsy in 2020 was > 75% and that the overall risk of inconclusive diagnoses was 7% (3% due to amplification failure and 4% due to poor quality of the molecular analysis). Nonetheless, high-quality centers published rates of inconclusive diagnoses lower than 3%, therefore chosen as benchmark value in this Consensus [70, 76, 77].

Finally, the identification of PIs during ovarian stimulation are important quantitative and qualitative measures of IQC, like FORT [78], that is significantly higher in women who achieved a pregnancy [73, 78]. Nevertheless, a low FORT (e.g., 30%) indicates hypo-response, due to the discrepancy between the relatively low number of pre-ovulatory follicles which develop following ovarian stimulation compared to the number of antral follicles available at its beginning [20]. The main limitation of FORT is the lack of feasibility in ultrasound scanning at the start of stimulation during routinary activity. Despite this, we believe that—as for late FOI—this measure could help to better assessing the quality of ovarian stimulation, with a low FORT reflecting clinicians’ inability to identifying the correct starting dose to elicit a good oocyte recruitment.

Lastly, CLBR is undoubtedly the most important indicator of IVF efficacy; it encompasses all steps of the journey and testifies whether the chance of each patients’ population is met without being affected by the clinical strategies and laboratory protocols adopted and/or by operators’ performance. This measure can be expressed per intention to treat, per started cycle, as well as cumulatively on multiple attempts in a longer timeframe. The longer the follow-up, the higher its insights. Nevertheless, SIFES-MR and SIERR experts decided to include the CLBR per started cycle only as a PI. Although recognizing its critical value, the data collection requires at least one year to accurately summarize the CLBR and many IVF centers, unfortunately, do not follow up the couples to that end, as they probably should. CLBR is in our view the most relevant outcome measure that should be assessed as part of any trial in IVF, and that should be used to guide patient counseling about their reproductive chance at each center; conversely, from a IQC perspective, its value is limited, in comparison to the KPIs included in our list, all more easily obtainable and actionable.

### *RIs: Statements with poor agreement (*< *40%)*

1. **Time between the infertility consultation and decision making.**

According to the National Institute for Health and Care Excellence [79] a couple in their reproductive age who has not conceived after 1 year of unprotected vaginal sexual intercourse, in the absence of known causes of infertility, should be offered infertility consultation to assess their fertility. However, when the woman is 36 years old or beyond, or there is a clinical known of infertility, or a history of factors affecting her fertility, specialistic consultation should be offered earlier. IVF counseling is crucial in the decision-making process to outline all possible options for attempting at a conception, but in the meantime coping with the effects and implications of what patients undertake. The time between the first consultation and the decision making is essential to maximize the future chance to conceive. A detailed and complete counseling, based on the couple's clinical history and on the possible options, is mandatory to accelerate the decision-making process, without neglecting the emotional component of the couple.

2. ***Time invested in the infertility work-up.***

Infertility work-up consists of a series of tests prescribed to the couple to identify a cause of infertility and outline a therapeutic strategy. Based on the numerous tests available, this step is crucial to reduce the time between diagnosis and treatment and to optimize the cost-effectiveness. IVF centers adopted different approaches of either a concise work-up or a complete screening.

3. ***Treatment discontinuation.***

Couples often discontinue their treatment without having achieved a pregnancy. The competence and benchmark values proposed were ≤ 50% for Minimum Expected and ≤ 25% for Best Practice Goal. However, it is very difficult and inappropriate to compare discontinuation rates between centers and countries, due to the heterogeneity of cost, reimbursement policies, accessibility to infertility services, etcetera [80, 81]. Reducing discontinuation rates is crucial to further improve the efficacy and cost-effectiveness of IVF treatments. Discontinuation should be considered an adverse outcome because early cessation of treatment prevents the couple from fulfilling their expected CLBR on a multi-cycle perspective, therefore impacting on the efficacy of the whole IVF journey. Discontinuation rates reported among couples undergoing IVF show a large variation from 20 to 60% depending on countries and centers within the same country [82, 83]. The main reasons for discontinuation are postponement of treatment, physical and psychological burden, relational and personal problems, treatment rejection, organizational and clinical issues [84]. All strategies that seek to reduce the discontinuation rate should be evaluated in all patients, but especially in very poor prognosis women (i.e., Bologna Criteria), and infertility counseling should be considered a critical step to make the patients aware of their realistic chance to conceive.

4. ***Prevalence of failed OPU***

This indicator is defined as the failure to retrieve oocytes during OPU, including empty follicle syndrome (EFS), despite apparently normal development of ovarian follicles and appropriate estradiol production by granulosa cells [85]. Two kinds of EFS have been described: (i) the ‘genuine’ form, which occurs after a correct ovulation trigger (by hCG or GnRH-analogue), and (ii) the ‘false’ form, which is associated with low hCG or LH levels and is imputable to trigger administration error or, for example, a result of rapid metabolic clearance in the patient. The total failure to retrieve oocytes represents a sporadic event rather than a true syndrome [85]. The competence and benchmark values were proposed as ≤ 7% for Minimum Expected and ≤ 0.5% for Best Practice Goal.

### KPIs in ART: a new formula to generate a unique comprehensive center performance score

A novelty of this Consensus is the proposal of a methodology to test the quality of each center based on the suggested KPIs. Indeed, the panel of experts suggested a value from 1 to 5 for each of these indicators to “weigh” them and outline a “weighted average” to include all parameters in a unique comprehensive value (Table 1). Each center should then outline a “score” from -1 to + 1 depending on their performance for all KPIs. If the performance is lower than the competence value for that KPI the “score” will be “-1”, if the performance is between the competence and benchmark values the “score” will be “0”, and if the performance is higher than the benchmark value the “score” will be “ + 1”. The result of the “weighted average” is a “CPS” calculated as described in the formula hereafter. The overall performance of the IVF center is graded as low if the “CPS” is < -0.5, average if between -0.5 and 0, good if between 0 and 0.5, and excellent if > 0.5.

**Table:** **Center Performance Score (CPS) formula.** $\mathrm{Weighted\;average\;(V=suggested\;Value,\;S=calculated\;Score,\;n=number\;of\;sub-categories\;reported\;for\;V1_{a,b},\;V7_{a,b},V8_{a,b,c,d}\;and\;V{10}_{a,b,c,d},\;respectively)}$

$$\begin{aligned}\frac{\begin{array}{c}\lbrack(\mathrm V1\ast\mathrm S1\mathrm a+\mathrm V1\ast\mathrm S1\mathrm b)/\mathrm n\rbrack+\mathrm V2\ast\mathrm S2+\mathrm V3\ast\mathrm S3+\mathrm V4\ast\mathrm S4+\mathrm V5\ast\mathrm S5+\mathrm V6\ast\mathrm S6+\lbrack(\mathrm V7\ast\mathrm S7\mathrm a+\mathrm V7\ast\mathrm S7\mathrm b)/\mathrm n\rbrack+\lbrack(\mathrm V8\ast\mathrm S8\mathrm a+\\\mathrm V8\ast\mathrm S8\mathrm b+\mathrm V8\ast\mathrm S8\mathrm c+\mathrm V8\ast\mathrm S8\mathrm d)/\mathrm n\rbrack+\mathrm V9\ast\mathrm S9+\lbrack(\mathrm V10\ast\mathrm S10\mathrm a+\mathrm V10\ast\mathrm S10\mathrm b+\mathrm V10\ast\mathrm S10\mathrm c+\mathrm V10\ast\mathrm S10\mathrm d)/\mathrm n\rbrack+\mathrm V11\ast\mathrm S11\end{array}}{\mathrm V1+\mathrm V2+\mathrm V3+\mathrm V4+\mathrm V5+\mathrm V6+\mathrm V7+\mathrm V8+\mathrm V9+\mathrm V10+\mathrm V11}\end{aligned}$$	

We provided two Excel files as Supplementary Material. The first file shows 3 examples, namely an excellent, an average and a poor CPS. The second file instead can be used by the readers to automatically calculate their CPS by simply adding the score -1, 0 or + 1 for each KPI and the total number of cycles performed. In both Excel files, the second sheets automatically generate a graph mirroring the CPS according to the number of cycles performed. In case an IVF center does not conduct blastocyst culture or ICSI, for instance, that value with its related score should be removed from both nominator and denominator. The same reasoning applies to the values and score for sub-categories such as PGT-A, for instance.

Of note, The CPS is mostly meant as a tool for IQC and performance self-assessment. Although its formula has been defined to also account for differences between patients’ populations across IVF centers, a comprehensive and accurate comparison between different clinics can be hardly conducted. The CLBR still remains in our view the main clinical measure of success in IVF, which should be complemented with a series of other indicators as recently proposed by Rienzi et al. [54]. Future prospective studies are invited to use the CPS. This consensus and the CPS formula will be subject to regular updates, whenever required based on users' feedback and upcoming clinical and laboratory advances in IVF.

## Conclusion

The clinical and laboratory advances in IVF profoundly changed the treatment of infertile couples, encouraging IVF specialists discussing which indicators are the most useful to assess all clinical steps in ART. In this regard, all the advances in the IVF laboratory, from blastocyst culture to vitrification, are aimed at an efficient clinical management, mandatory to help our patients fulfilling their predetermined chance of success. The KPIs, PIs and RIs proposed in this Italian consensus include several essential steps of a modern IVF clinic, encompassing both clinical and embryological aspects. The identification of sharable KPIs, PIs and RIs in IVF is a very difficult task due to specific settings (private/public), different regulation, skills, IVF laboratory efficiency, etcetera. Embryologists and clinicians must communicate regularly and partner effectively to improve IVF efficacy and efficiency. This Italian Consensus involved both clinical and laboratory perspectives to generate a comprehensive score indicative of an all-round assessment of the clinics.

## Supplementary Information

Below is the link to the electronic supplementary material.

Supplementary file1 (XLSX 20.3 KB)

Supplementary file2 (XLSX 18.5 KB)

## Data Availability

Data sharing is not applicable to this article as no new data were created or analyzed in this study.

## References

[1] Ravitsky V, Kimmins S (2019). The forgotten men: rising rates of male infertility urgently require new approaches for its prevention, diagnosis and treatment. Biol Reprod.

[2] c A, Mulgund A, Hamada A, Chyatte MR (2015). A unique view on male infertility around the globe. Reprod Biol Endocrinol..

[3] Annual Capri Workshop Group (2020). IVF, from the past to the future: the inheritance of the Capri Workshop Group. Hum Reprod Open.

[4] Jain T, Grainger D, Ball G, Gibbons W, Rebar R, Robins J, Leach R. 30 years of data: impact of the United States in vitro fertilization data registry on advancing fertility care. Fertility and Sterility. 2019;111. 10.1016/j.fertnstert.2018.11.015. 10.1016/j.fertnstert.2018.11.015 30737003

[5] Vlaisavljevic V, Apter S, Capalbo A, D'Angelo A, Gianaroli L, Griesinger G, et al. The Maribor consensus: report of an expert meeting on the development of performance indicators for clinical practice in ART. Hum Reprod Open. 2021;2021(3):hoab022. 10.1093/hropen/hoab022. 10.1093/hropen/hoab022 PMC8254491 34250273

[6] Wilkinson J, Roberts SA, Showell M, Brison DR, Vail A (2016). No common denominator: a review of outcome measures in IVF RCTs. Hum Reprod.

[7] Dyer S, Chambers GM, de Mouzon J, Nygren KG, Zegers-Hochschild F, Mansour R, et al. International Committee for Monitoring Assisted Reproductive Technologies world report: Assisted Reproductive Technology 2008, 2009 and 2010. Hum Reprod. 2016;31(7):1588–609. 10.1093/humrep/dew082. 10.1093/humrep/dew082 27207175

[8] Rienzi L, Gracia C, Maggiulli R, LaBarbera AR, Kaser DJ, Ubaldi FM (2017). Oocyte, embryo and blastocyst cryopreservation in ART: systematic review and meta-analysis comparing slow-freezing versus vitrification to produce evidence for the development of global guidance. Hum Reprod Update.

[9] Medicine ASIR, Embryology. ESIG. Istanbul consensus workshop on embryo assessment: proceedings of an expert meeting. Reprod Biomed Online. 2011: 632–46 10.1016/j.rbmo.2011.02.001 21481639

[10] Fabozzi G, Cimadomo D, Maggiulli R, Vaiarelli A, Ubaldi FM, Rienzi L. Which key performance indicators are most effective in evaluating and managing an in vitro fertilization laboratory? Fertil Steril. 2020;114(1):9–15. 10.1016/j.fertnstert.2020.04.054. 10.1016/j.fertnstert.2020.04.054 32532495

[11] coticchio.biogenesi@grupposandonato.it ESIGoEaASiRMEa. The Vienna consensus: report of an expert meeting on the development of ART laboratory performance indicators. Reprod Biomed Online. 2017;35(5):494–510. 10.1016/j.rbmo.2017.06.015. 10.1016/j.rbmo.2017.06.015 28784335

[12] Alviggi C, Humaidan P, Howles CM, Tredway D, Hillier SG. Biological versus chronological ovarian age: implications for assisted reproductive technology. Reprod Biol Endocrinol. 2009;7:101. 10.1186/1477-7827-7-101. 10.1186/1477-7827-7-101 PMC2764709 19772632

[13] Conforti A, Esteves SC, Humaidan P, Longobardi S, D'Hooghe T, Orvieto R, et al. Recombinant human luteinizing hormone co-treatment in ovarian stimulation for assisted reproductive technology in women of advanced reproductive age: a systematic review and meta-analysis of randomized controlled trials. Reprod Biol Endocrinol. 2021;19(1):91. 10.1186/s12958-021-00759-4. 10.1186/s12958-021-00759-4 PMC8215738 34154604

[14] Committee ACoOaGCoGPaP. Female age-related fertility decline. Committee Opinion No. 589. Fertil Steril. 2014;101(3):633–4. 10.1016/j.fertnstert.2013.12.032. 10.1016/j.fertnstert.2013.12.032 24559617

[15] Esteves SC, Conforti A, Sunkara SK, Carbone L, Picarelli S, Vaiarelli A, et al. Improving Reporting of Clinical Studies Using the POSEIDON Criteria: POSORT Guidelines. Front Endocrinol (Lausanne). 2021;12:587051. 10.3389/fendo.2021.587051. 10.3389/fendo.2021.587051 PMC8017440 33815269

[16] La Marca A, Sunkara SK (2014). Individualization of controlled ovarian stimulation in IVF using ovarian reserve markers: from theory to practice. Hum Reprod Update.

[17] Calhaz-Jorge C, De Geyter CH, Kupka MS, Wyns C, Mocanu E, Motrenko T, et al. Survey on ART and IUI: legislation, regulation, funding and registries in European countries: The European IVF-monitoring Consortium (EIM) for the European Society of Human Reproduction and Embryology (ESHRE). Hum Reprod Open. 2020;2020(1):hoz044. 10.1093/hropen/hoz044. 10.1093/hropen/hoz044 PMC7002185 32042927

[18] Kushnir VA, Vidali A, Barad DH, Gleicher N. The status of public reporting of clinical outcomes in assisted reproductive technology. Fertil Steril. 2013;100(3):736–41. 10.1016/j.fertnstert.2013.05.012. 10.1016/j.fertnstert.2013.05.012 23755956

[19] Wyns C, Bergh C, Calhaz-Jorge C, De Geyter C, Kupka MS, Motrenko T, et al. ART in Europe, 2016: results generated from European registries by ESHRE. Hum Reprod Open. 2020;2020(3):hoaa032. 10.1093/hropen/hoaa032. 10.1093/hropen/hoaa032 PMC7394132 32760812

[20] Alviggi C, Conforti A, Esteves SC, Vallone R, Venturella R, Staiano S, et al. Understanding Ovarian Hypo-Response to Exogenous Gonadotropin in Ovarian Stimulation and Its New Proposed Marker-The Follicle-To-Oocyte (FOI) Index. Front Endocrinol (Lausanne). 2018;9:589. 10.3389/fendo.2018.00589. 10.3389/fendo.2018.00589 PMC6199413 30386293

[21] Chen L, Wang H, Zhou H, Bai H, Wang T, Shi W, et al. Follicular Output Rate and Follicle-to-Oocyte Index of Low Prognosis Patients According to POSEIDON Criteria: A Retrospective Cohort Study of 32,128 Treatment Cycles. Front Endocrinol (Lausanne). 2020;11:181. 10.3389/fendo.2020.00181. 10.3389/fendo.2020.00181 PMC7154057 32318023

[22] Conforti A, Esteves SC, Cimadomo D, Vaiarelli A, Di Rella F, Ubaldi FM (2019). Management of Women With an Unexpected Low Ovarian Response to Gonadotropin. Front Endocrinol (Lausanne).

[23] Poulain M, Younes R, Pirtea P, Trichereau J, de Ziegler D, Benammar A, et al. Impact of Ovarian Yield-Number of Total and Mature Oocytes Per Antral Follicular Count-On Live Birth Occurrence After IVF Treatment. Front Med (Lausanne). 2021;8:702010. 10.3389/fmed.2021.702010. 10.3389/fmed.2021.702010 PMC8421602 34504852

[24] The ESHRE Guideline Group on Ovarian Stimulation, Bosch E, Broer S, Griesinger G, Grynberg M, Humaidan P, Kolibianakis E, Kunicki M, La Marca A, Lainas G, Le Clef N, Massin N, Mastenbroek S, Polyzos N, Sunkara SK, Timeva T, Töyli M, Urbancsek J, Vermeulen N, Broekmans F. ESHRE guideline: ovarian stimulation for IVF/ICSI†. Hum Reprod Open. 2020;(2):hoaa009. 10.1093/hropen/hoaa009. 10.1093/hropen/hoaa009 PMC7203749 32395637

[25] Parrella A, Irani M, Keating D, Chow S, Rosenwaks Z, Palermo GD (2019). High proportion of immature oocytes in a cohort reduces fertilization, embryo development, pregnancy and live birth rates following ICSI. Reprod BioMed Online.

[26] Beall S, Brenner C, Segars J. Oocyte maturation failure: a syndrome of bad eggs. Fertil Steril. 2010;94(7):2507–13. 10.1016/j.fertnstert.2010.02.037. 10.1016/j.fertnstert.2010.02.037 PMC2946974 20378111

[27] ESHRE Working Group on Ultrasound in ART, D’Angelo A, Panayotidis C, Amso N, Marci R, Matorras R, Onofriescu M, Turp AB, Vandekerckhove F, Veleva Z, Vermeulen N, Vlaisavljevic V. Recommendations for good practice in ultrasound: oocyte pick up†. Hum Reprod Open. 2019;2019(4):hoz025. 10.1093/hropen/hoz025. 10.1093/hropen/hoz025 PMC6903452 31844683

[28] Levi-Setti PE, Cirillo F, Scolaro V, Morenghi E, Heilbron F, Girardello D, et al. Appraisal of clinical complications after 23,827 oocyte retrievals in a large assisted reproductive technology program. Fertil Steril. 2018;109(6):1038–43.e1. 10.1016/j.fertnstert.2018.02.002. 10.1016/j.fertnstert.2018.02.002 29871795

[29] Maxwell KN, Cholst IN, Rosenwaks Z. The incidence of both serious and minor complications in young women undergoing oocyte donation. Fertil Steril. 2008;90(6):2165–71. 10.1016/j.fertnstert.2007.10.065. 10.1016/j.fertnstert.2007.10.065 18249368

[30] Bodri D, Guillen JJ, Polo A, Trullenque M, Esteve C, Coll O (2008). Complications related to ovarian stimulation and oocyte retrieval in 4052 oocyte donor cycles. Reprod Biomed Online.

[31] Rubino P, Vigano P, Luddi A, Piomboni P (2016). The ICSI procedure from past to future: a systematic review of the more controversial aspects. Hum Reprod Update.

[32] Mazzilli R, Vaiarelli A, Dovere L, Cimadomo D, Ubaldi N, Ferrero S, et al. Severe male factor in in vitro fertilization: definition, prevalence, and treatment. An update. Asian J Androl. 2021. 10.4103/aja.aja_53_21. 10.4103/aja.aja_53_21 PMC8887096 34259196

[33] Medicine ASIR. The Alpha consensus meeting on cryopreservation key performance indicators and benchmarks: proceedings of an expert meeting. Reprod Biomed Online. 2012;25(2):146–67. 10.1016/j.rbmo.2012.05.006. 10.1016/j.rbmo.2012.05.006 22727888

[34] Zacà C, Coticchio G, Vigiliano V, Lagalla C, Nadalini M, Tarozzi N, et al. Fine-tuning IVF laboratory key performance indicators of the Vienna consensus according to female age. J Assist Reprod Genet. 2022;39(4):945–52. 10.1007/s10815-022-02468-2. 10.1007/s10815-022-02468-2 PMC9050984 35338418

[35] Cai QF, Wan F, Huang R, Zhang HW. Factors predicting the cumulative outcome of IVF/ICSI treatment: a multivariable analysis of 2450 patients. Hum Reprod. 2011;26(9):2532–40. 10.1093/humrep/der228. 10.1093/humrep/der228 21771773

[36] Rosen MP, Shen S, Rinaudo PF, Huddleston HG, McCulloch CE, Cedars MI. Fertilization rate is an independent predictor of implantation rate. Fertil Steril. 2010;94(4):1328–33. 10.1016/j.fertnstert.2009.05.024. 10.1016/j.fertnstert.2009.05.024 19560757

[37] Scaravelli G, Zacà C, Levi Setti PE, Livi C, Ubaldi FM, Villani MT, et al. Fertilization rate as a novel indicator for cumulative live birth rate: a multicenter retrospective cohort study of 9,394 complete in vitro fertilization cycles. Fertil Steril. 2021;116(3):766–73. 10.1016/j.fertnstert.2021.04.006. 10.1016/j.fertnstert.2021.04.006 33972085

[38] Wang J, Diao Z, Fang J, Zhu L, Xu Z, Lin F, et al. The influence of day 3 embryo cell number on the clinical pregnancy and live birth rates of day 5 single blastocyst transfer from frozen embryo transfer cycles. BMC Pregnancy Childbirth. 2022;22(1):980. 10.1186/s12884-022-05337-z. 10.1186/s12884-022-05337-z PMC9798545 36581843

[39] Jones CA, Acharya KS, Acharya CR (2020). Patient and in vitro fertilization (IVF) cycle characteristics associated with variable blastulation rates: a retrospective study from the Duke Fertility Center (2013–2017). Middle East Fertil Soc J.

[40] Irani M, O'Neill C, Palermo GD, Xu K, Zhang C, Qin X, et al. Blastocyst development rate influences implantation and live birth rates of similarly graded euploid blastocysts. Fertil Steril. 2018;110(1):95–102.e1. 10.1016/j.fertnstert.2018.03.032. 10.1016/j.fertnstert.2018.03.032 29908774

[41] Maggiulli R, Cimadomo D, Fabozzi G, Papini L, Dovere L, Ubaldi FM (2020). The effect of ICSI-related procedural timings and operators on the outcome. Hum Reprod.

[42] Romanski PA, Aluko A, Bortoletto P, Elias R, Rosenwaks Z. Age-specific blastocyst conversion rates in embryo cryopreservation cycles. Reprod Biomed Online. 2022;45(3):432–9. 10.1016/j.rbmo.2022.04.006. 10.1016/j.rbmo.2022.04.006 35610153

[43] Park SU, Walsh L, Berkowitz KM. Mechanisms of ovarian aging. Reproduction. 2021;162(2):R19-R33. 10.1530/REP-21-0022. 10.1530/REP-21-0022 PMC9354567 33999842

[44] Vassena R, Boué S, González-Roca E, Aran B, Auer H, Veiga A, et al. Waves of early transcriptional activation and pluripotency program initiation during human preimplantation development. Development. 2011;138(17):3699–709. 10.1242/dev.064741. 10.1242/dev.064741 PMC4074286 21775417

[45] Cimadomo D, Sosa Fernandez L, Soscia D, Fabozzi G, Benini F, Cesana A, et al. Inter-centre reliability in embryo grading across several IVF clinics is limited: implications for embryo selection. Reprod Biomed Online. 2021;44(1):39–48. 10.1016/j.rbmo.2021.09.022. 10.1016/j.rbmo.2021.09.022 34819249

[46] Corti L, Cermisoni GC, Alteri A, Pagliardini L, Ambrosini G, Andrisani A, et al. Clinical Outcomes Deriving from Transfer of Blastocysts Developed in Day 7: a Systematic Review and Meta-Analysis of Frozen-Thawed IVF Cycles. Reprod Sci. 2021. 10.1007/s43032-020-00424-y. 10.1007/s43032-020-00424-y 33449349

[47] Apter S, Ebner T, Freour T, Guns Y, Kovacic B, Le Clef N, et al. Eshre Working group on Time-lapse technology: Good practice recommendations for the use of time-lapse technology. Hum Reprod Open. 2020;2020(2):hoaa008. 10.1093/hropen/hoaa008. 10.1093/hropen/hoaa008 PMC7081060 32206731

[48] Zegers-Hochschild F, Adamson GD, Dyer S, Racowsky C, de Mouzon J, Sokol R (2017). The International Glossary on Infertility and Fertility Care, 2017. Fertil Steril.

[49] Braakhekke M, Kamphuis EI, Dancet EA, Mol F, van der Veen F, Mol BW (2014). Ongoing pregnancy qualifies best as the primary outcome measure of choice in trials in reproductive medicine: an opinion paper. Fertil Steril.

[50] Cimadomo D, Capalbo A, Dovere L, Tacconi L, Soscia D, Giancani A (2021). Leave the past behind: women's reproductive history shows no association with blastocysts' euploidy and limited association with live birth rates after euploid embryo transfers. Hum Reprod.

[51] Cimadomo D, Fabozzi G, Dovere L, Maggiulli R, Albricci L, Innocenti F, et al. Clinical, obstetric and perinatal outcomes after vitrified-warmed euploid blastocyst transfer are independent of cryo-storage duration. Reprod Biomed Online. 2021. 10.1016/j.rbmo.2021.09.027. 10.1016/j.rbmo.2021.09.027 34862135

[52] Glujovsky D, Farquhar C, Quinteiro Retamar AM, Alvarez Sedo CR, Blake D. Cleavage stage versus blastocyst stage embryo transfer in assisted reproductive technology. Cochrane Database Syst Rev. 2016;(6):CD002118. 10.1002/14651858.CD002118.pub5. 10.1002/14651858.CD002118.pub5 27357126

[53] Reig A, Franasiak J, Scott RT, Jr., Seli E. The impact of age beyond ploidy: outcome data from 8175 euploid single embryo transfers. J Assist Reprod Genet. 2020;37(3):595–602. 10.1007/s10815-020-01739-0. 10.1007/s10815-020-01739-0 PMC7125286 32173784

[54] Rienzi L, Cimadomo D, Vaiarelli A, Gennarelli G, Holte J, Livi C, et al. Measuring success in IVF is a complex multidisciplinary task: time for a consensus? Reprod Biomed Online. 2021;43(5):775–8. 10.1016/j.rbmo.2021.08.012. 10.1016/j.rbmo.2021.08.012 34493463

[55] Smithers PR, Halliday J, Hale L, Talbot JM, Breheny S, Healy D (2003). High frequency of cesarean section, antepartum hemorrhage, placenta previa, and preterm delivery in in-vitro fertilization twin pregnancies. Fertil Steril.

[56] Romanenko THSO, Ovcharenko SO (2021). A statistical analysis of obstetric and perinatal complications in singleton and multiple pregnancies once assisted reproductive technologies are used. Wiadomości Lekarskie.

[57] Ubaldi FM, Capalbo A, Colamaria S, Ferrero S, Maggiulli R, Vajta G (2015). Reduction of multiple pregnancies in the advanced maternal age population after implementation of an elective single embryo transfer policy coupled with enhanced embryo selection: pre- and post-intervention study. Hum Reprod.

[58] Vega M, Zaghi S, Buyuk E, Jindal S (2018). Not all twins are monozygotic after elective single embryo transfer: analysis of 32,600 elective single embryo transfer cycles as reported to the Society for Assisted Reproductive Technology. Fertil Steril.

[59] Riishede IBWC, Kvist Ekelund C, Pinborg A, Tabor A. Risk of miscarriage in women conceiving after medically assisted reproduction (MAR) with an ultrasound verified viable pregnancy at 6-8 weeks of gestation. Reprod BioMed Online. 39. 10.1016/j.rbmo.2019.06.010. 10.1016/j.rbmo.2019.06.010 31628037

[60] Farr SL, Schieve LA, Jamieson DJ. Pregnancy loss among pregnancies conceived through assisted reproductive technology, United States, 1999–2002. Am J Epidemiol. 2007;165(12):1380–8. 10.1093/aje/kwm035. 10.1093/aje/kwm035 17351291

[61] Heffner LJ (2004). Advanced maternal age–how old is too old?. N Engl J Med.

[62] Arredondo F, Noble LS (2006). Endocrinology of recurrent pregnancy loss. Semin Reprod Med.

[63] NO, Green-top Guideline. The management of ovarian hyperstimulation syndrome. 2016. 10.1111/1471-0528.70195 41975565

[64] Al-Inany HG, Youssef MA, Ayeleke RO, Brown J, Lam WS, Broekmans FJ. Gonadotrophin-releasing hormone antagonists for assisted reproductive technology. Cochrane Database Syst Rev. 2016;4:CD001750. 10.1002/14651858.CD001750.pub4. 10.1002/14651858.CD001750.pub4 PMC8626739 27126581

[65] Papanikolaou EG, Tournaye H, Verpoest W, Camus M, Vernaeve V, Van Steirteghem A, et al. Early and late ovarian hyperstimulation syndrome: early pregnancy outcome and profile. Hum Reprod. 2005;20(3):636–41. 10.1093/humrep/deh638. 10.1093/humrep/deh638 15576388

[66] Griesinger G, Verweij PJ, Gates D, Devroey P, Gordon K, Stegmann BJ, et al. Prediction of Ovarian Hyperstimulation Syndrome in Patients Treated with Corifollitropin alfa or rFSH in a GnRH Antagonist Protocol. PLoS One. 2016;11(3):e0149615. 10.1371/journal.pone.0149615. 10.1371/journal.pone.0149615 PMC4780699 26950065

[67] Toftager M, Bogstad J, Bryndorf T, Lossl K, Roskaer J, Holland T (2016). Risk of severe ovarian hyperstimulation syndrome in GnRH antagonist versus GnRH agonist protocol: RCT including 1050 first IVF/ICSI cycles. Hum Reprod.

[68] Vlaisavljević V, Kovačič B, Knez J. Cumulative live birth rate after GnRH agonist trigger and elective cryopreservation of all embryos in high responders. Reprod Biomed Online. 2017;35(1):42–8. 10.1016/j.rbmo.2017.03.017. 10.1016/j.rbmo.2017.03.017 28416291

[69] De Rycke M, Belva F, Goossens V, Moutou C, SenGupta SB, Traeger-Synodinos J, et al. ESHRE PGD Consortium data collection XIII: cycles from January to December 2010 with pregnancy follow-up to October 2011. Hum Reprod. 2015;30(8):1763–89. 10.1093/humrep/dev122. 10.1093/humrep/dev122 26071418

[70] Cimadomo D, Rienzi L, Romanelli V, Alviggi E, Levi-Setti PE, Albani E (2018). Inconclusive chromosomal assessment after blastocyst biopsy: prevalence, causative factors and outcomes after re-biopsy and re-vitrification. A multicenter experience Hum Reprod.

[71] Neal SA, Forman EJ, Juneau CR, Morin J, Molinaro T, Sun L, et al. Rebiopsy and preimplantation genetic screening (PGS) reanalysis for embryos with an initial non-diagnostic result yields a euploid result in the majority of cases. Fertil Steril. 2017;108(3):e276. 10.1016/j.fertnstert.2017.07.821.

[72] Genro VK GM, Scheffer JB, Roux I, Frydman R, Fanchin R. Serum anti-Müllerian hormone levels are negatively related to Follicular Output RaTe (FORT) in normo-cycling women undergoing controlled ovarian hyperstimulation. Hum Reprod. 2011:671–7. 10.1093/humrep/deq361 21177311

[73] Gallot V, Berwanger da Silva AL, Genro V, Grynberg M, Frydman N, Fanchin R. Antral follicle responsiveness to follicle-stimulating hormone administration assessed by the Follicular Output RaTe (FORT) may predict in vitro fertilization-embryo transfer outcome. Hum Reprod. 2012;27(4):1066–72. 10.1093/humrep/der479. 10.1093/humrep/der479 22279090

[74] Malizia BA, Hacker MR, Penzias AS (2009). Cumulative live-birth rates after in vitro fertilization. N Engl J Med.

[75] Luke B, Brown MB, Wantman E, Lederman A, Gibbons W, Schattman GL (2012). Cumulative birth rates with linked assisted reproductive technology cycles. N Engl J Med.

[76] Neal SA, Sun L, Jalas C, Morin SJ, Molinaro TA, Scott RT (2019). When next-generation sequencing-based preimplantation genetic testing for aneuploidy (PGT-A) yields an inconclusive report: diagnostic results and clinical outcomes after re biopsy. J Assist Reprod Genet.

[77] Capalbo A, Ubaldi FM, Cimadomo D, Maggiulli R, Patassini C, Dusi L (2016). Consistent and reproducible outcomes of blastocyst biopsy and aneuploidy screening across different biopsy practitioners: a multicentre study involving 2586 embryo biopsies. Hum Reprod.

[78] Zhang N, Hao CF, Zhuang LL, Liu XY, Gu HF, Liu S, et al. Prediction of IVF/ICSI outcome based on the follicular output rate. Reprod Biomed Online. 2013;27(2):147–53. 10.1016/j.rbmo.2013.04.012. 10.1016/j.rbmo.2013.04.012 23768619

[79] For Women’s, National Collaborating Centre; Children’s Health, U. K. Fertility: Assessment and treatment for people with fertility problems. 2013.

[80] Brandes M, van der Steen JO, Bokdam SB, Hamilton CJ, de Bruin JP, Nelen WL (2009). When and why do subfertile couples discontinue their fertility care? A longitudinal cohort study in a secondary care subfertility population. Hum Reprod.

[81] Roest J, Van Heusden AM, Zeilmaker GH, Verhoeff A (1998). Treatment policy after poor fertilization in the first IVF cycle. J Assist Reprod Genet.

[82] Domar AD, Smith K, Conboy L, Iannone M, Alper M. A prospective investigation into the reasons why insured United States patients drop out of in vitro fertilization treatment. Fertil Steril. 2010;94(4):1457–9. 10.1016/j.fertnstert.2009.06.020. 10.1016/j.fertnstert.2009.06.020 19591985

[83] Van den Broeck U, Holvoet L, Enzlin P, Bakelants E, Demyttenaere K, D'Hooghe T (2009). Reasons for dropout in infertility treatment. Gynecol Obstet Invest.

[84] Gameiro S, Boivin J, Peronace L, Verhaak CM (2012). Why do patients discontinue fertility treatment? A systematic review of reasons and predictors of discontinuation in fertility treatment. Hum Reprod Update.

[85] Coulam CB, Bustillo M, Schulman JD (1986). Empty follicle syndrome. Fertil Steril.

